# Prevalence of MMTV-like sequences in breast cancer samples in Romanian patients-there is a geographic difference compared to the Western world

**DOI:** 10.1186/s13027-023-00486-y

**Published:** 2023-06-20

**Authors:** Zsolt Fekete, Bristena Octavia Tertan, Lajos Raduly, Dan Tudor Eniu, Rares Buiga, Mihaela Galatar, Ioana Berindan-Neagoe

**Affiliations:** 1 Oncology-Radiotherapy, Department of Oncology, Iuliu Hațieganu University of Medicine and Pharmacy, Cluj-Napoca, Romania; 2 Institute of Oncology “Prof Dr. Ion Chiricuță“, Radiotherapy III, Cluj-Napoca, Romania; 3 “Iuliu Hațieganu” University of Medicine and Pharmacy, Cluj-Napoca, Romania; 4 Research Center for Functional Genomics, Biomedicine and Translational Medicine, Iuliu Hațieganu University of Medicine and Pharmacy, Cluj-Napoca, Romania; 5 Oncological Surgery and Gynecologic Oncology, Department of Oncology, “Iuliu Hațieganu” University of Medicine and Pharmacy, Cluj-Napoca, Romania; 6 Surgery Department, Institute of Oncology “Prof Dr. Ion Chiricuță“, Cluj-Napoca, Romania; 7 Pathology Department, Institute of Oncology “Prof Dr. Ion Chiricuță“, Cluj-Napoca, Romania

**Keywords:** Mouse mammary tumor virus (MMTV), Breast cancer, Infectious disease, Epidemiology, Zoonosis

## Abstract

**Background:**

Breast cancer, although the most frequently diagnosed malignant tumor in humans, has a less clear etiology compared to other frequent cancer types. Mouse-mammary tumor virus (MMTV) is involved in breast cancer in mice and dogs and might play a role in the etiology of some breast cancers in humans, since an MMTV-like sequence was identified in 20–40% of breast cancer samples in Western Europe, USA, Australia and some other parts of the world. The purpose of our study was to identify MMTV-like DNA sequences in breast tissue samples from breast cancer patients who underwent curative surgery in our regional academic center in Romania, EU.

**Methods:**

We selected 75 patients with non-metastatic breast cancer treated surgically with curative intent, who did not undergo any neoadjuvant treatment. Out of these patients, 50 underwent radical lumpectomy and 25 modified radical mastectomy. Based on previous reports in the literature we searched using PCR the MMTV-like DNA *env* sequence in the breast cancer tissue and normal breast tissue obtained from the same patients.

**Results:**

None of the examined samples was positive for MMTV-like target sequences on PCR.

**Conclusions:**

We could not prove that MMTV plays a role in the etiology of breast cancer in our patient group. This finding is similar to those from publications of other geographically related research groups.

**Supplementary Information:**

The online version contains supplementary material available at 10.1186/s13027-023-00486-y.

## Background

In 2020, female breast cancer has surpassed lung cancer as the most commonly diagnosed cancer, with an estimated 2.3 million new cases (11.7%), being also the leading cause of mortality due to cancer in females around the world. Among women, breast cancer accounts for 1 in 4 cancer cases and for 1 in 6 cancer deaths. Incidence rates are 88% higher in transitioned countries than in transitioning countries with the highest incidence rates (> 80 per 100,000) in Australia/New Zealand, Western Europe (Belgium has the world’s highest incidence), Northern America, and Northern Europe and the lowest rates (< 40 per 100,000) in Central America, Eastern and Middle Africa, and South-Central Asia [1]. This pattern of distribution is thus far explained by different life-expectancy and various lifestyle factors.

Although there are various risk factors known for breast cancer, only about 10% of the cases have a precise genetic cofactor. Breast cancer associated gene 1 and 2 (BRCA1 and BRCA2) are two anti-oncogenes located on chromosome 17q21 and 13q12, respectively, and both encode tumor suppressor proteins. Totally, about 20–25% of hereditary breast cancers and 5–10% of all breast cancers are caused by BRCA1/2 mutations [2]. Family history of breast cancer, without a proven predisposing mutation, is another major risk factor [3]. For example women with a family history of breast cancer (two or more cases in women younger than 50 years or three or more cases at any age) who do not present BRCA mutations are approximately 11 times more likely to develop breast cancer [4]. Exogenous hormone use, such as contraceptives [5], ovulation-stimulating drugs [6] and some schedules of menopausal hormone replacement therapy [7] might also increase the risk of breast cancer or cause an earlier onset of the disease. There is much debate if hormones are true risk factors or just stimulate breast cancers that are formed without hormonal promotion at the very beginning. Other risk factors include obesity, alcohol consumption [8], radiation exposure or nulliparity [2].

One retrovirus that has been used for many years for the study of cancer pathogenesis in mice is the Mouse Mammary Tumor Virus (MMTV). It was discovered in 1936 as a milk-transmitted agent in some mice strains. The agent was then shown to have reverse transcriptase activity, similar to other retroviruses, and hormone-responsive elements in the viral genome that enhance viral replication during pregnancy [9]. In 1989 Harold Varmus was awarded the Nobel Prize for the observation that the insertion of MMTV proviral genome in host DNA resulted in the activation on proto-oncogenes.

The majority of breast cancers in mice are caused by MMTV [10].

Several studies examined the association between MMTV and human breast cancer. A major breakthrough came in 1972 with the identification of RNA in human breast cancer that was homologous to MMTV RNA [11]. Prior to the widespread use of PCR, MMTV-related sequences in human breast cancer cells were identified by hybridization techniques. Using hybridization methods Szakacs and Moscinski [12] identified in DNA sequences homologous to the entire MMTV provirus using LTR- long terminal repeat, *gag*, *pol* and *env* probes in 7 (13%) of 52 human breast cancers. However, it was difficult to distinguish MMTV gene sequences from those of the human endogenous retrovirus (HERV). HERV gene sequences are very similar to MMTV and may be the remnants of MMTV viruses that have become integrated into the human genome over millennia [13]. This problem was overcome by the Beatriz Pogo group by their identification of MMTV envelope gene sequences which were unique to MMTV [14].

Using PCR techniques directed at a 660 bp highly conserved portion of the MMTV-env gene with only 16% homology to the prototype HERV-K10 human endogenous retrovirus, Wang et al. were able to demonstrate MMTV- env specific sequences in 38.5% of the 314 breast carcinomas and in 6.9% of the 29 breast fibroadenoma samples, compared to only 1.8% of 107 samples of normal breast reduction mammoplasty tissues [15]. A series of similar studies using PCR primers and nested primers were then conduced in an attempt to correlate the presence of the MMTV specific 660 bp env sequence with mammary tumorigenesis. A meta-analysis published in 2019 analyzed 20 studies reported in 17 publications in which PCR was used to detect MMTV specific region identified by Wang et al.; 11 studies showed a positive correlation between the presence of the indicative MMTV signal and breast tumor tissue at the *p* < 0.01 significance level. To further investigate the presence of a sub-fragment of the highly conserved MMTV *env* region, laser microdissection techniques were used to study breast cancer epithelial cells followed by real-time PCR analysis [16]. MMTV was identified in 82% of ductal carcinoma in situ specimens and 35% of invasive ductal carcinoma (currently carcinoma NOS) specimens compared to no identification in normal breast specimens from reduction mammoplasty.

The aim of our study was to identify MMTV *env* viral sequences in surgical breast cancer tissue samples in Romania, to verify in this part of South-Eastern Europe if there is a possibility that some breast cancers are related to MMTV infection or harbor MMTV-like sequences.

## Methods

We conducted a retrospective study in which we analyzed the presence of the MMTV *env* gene sequence in human breast cancer samples. The samples were collected from the tissue archive of the Oncological Institute of Cluj-Napoca. They were FFPE breast tissue samples obtained from either mastectomies or lumpectomies of women diagnosed with invasive ductal carcinoma between 2003 and 2011. The patients selected for the study did not undergo neoadjuvant chemo- or radiotherapy.

For each patient, a pathologist (R.B.) examined the tissue samples and selected one sample that contained tumor tissue characteristic for breast carcinoma NOS (not otherwise specified) and one sample that contained normal peri-tumoral tissue. The formalin-fixed paraffin-embedded samples were each sectioned at 10 µm and 5–6 sections were put into a tube, two tubes for each tumor sample and two tubes for each normal tissue sample, therefore 4 tubes corresponding to each patient.

DNA was extracted from deparaffinized sections of each paraffin block using PureLink® Genomic DNA Kit (Invitrogen), K182001, as instructed by the manufacturer.

The PCR reaction for the selected samples was performed with Thermo Scientific Phusion High-Fidelity PCR Master Mix according to the recommended protocol from a 200 ng total concentration of DNA. The condition used for polymerase chain reaction was 98 °C/30 s for initial denaturation, 98 °C/10 s, 61 °C/30 s and 72 °C/30 s for denaturation annealing and extension and 72 °C/10 min., 4 °C/hold for final extension in a Veriti™ 96-Well Thermal Cycler (ThermoFisher Scientific). Positive and negative controls were included in each run.

For the electrophoresis a 3% agarose gel was used with a ThermoFisher Scientific GeneRuler Low Range DNA ladder.

A 104 base pair oligonucleotide was ordered based on the gene bank MMTV-like virus envelope protein [ACCESSION #: GU109516] and was used as a positive control. This was **identical** to the oligonucleotide used by Tabriz et al. in Iran [17].

The primer used was MMTV env gPr73 [Mouse mammary tumor virus] forward, 5′-GATGGTATGAAGCAGGATGG-3′ and reverse, 5′-CCTCTTTTCTCTATATCTATTAGCTGAGGTAATC-3′. The primers used were **identical** to those used by Naccarato et al. in Italy [18].

## Results

### Patients

We selected 75 patients with ages between 32 and 77 years old; 25 patients underwent modified radical mastectomy and 50 patients were treated with lumpectomy. The characteristics of the patients and the descriptors of the tumors are summarized in Table 1.

**Table 1:** Patients’ characteristics

Variable	Number	%
Mean age (years)	54.29	
*Stage*		
IA	17	22.67
IIA	31	41.33
IIB	11	14.67
IIIA	5	6.67
IIIB	3	4
IIIC	8	10.66
*Histologic grade (Nottingham score)*		
I	13	17.33
II	23	30.66
III	39	52
*Estrogen receptors*		
Positive	58	77.33
Negative	17	22.66
*Progesterone receptors*		
Positive	53	70.66
Negative	22	29.33
*HER-2 receptors*		
Positive	29	38.66
Negative	46	61.33
Triple negative	16	21.33

Regarding the tumor (T) classification of the TNM staging system for the breast cancer, out of the 75 patients with invasive ductal carcinoma selected in our study, 16 were stage T1, 46 were T2, 4 were T3, 9 were T4. None of the tumors were classified as T4d—inflammatory breast cancer.

**Fig. 1:**
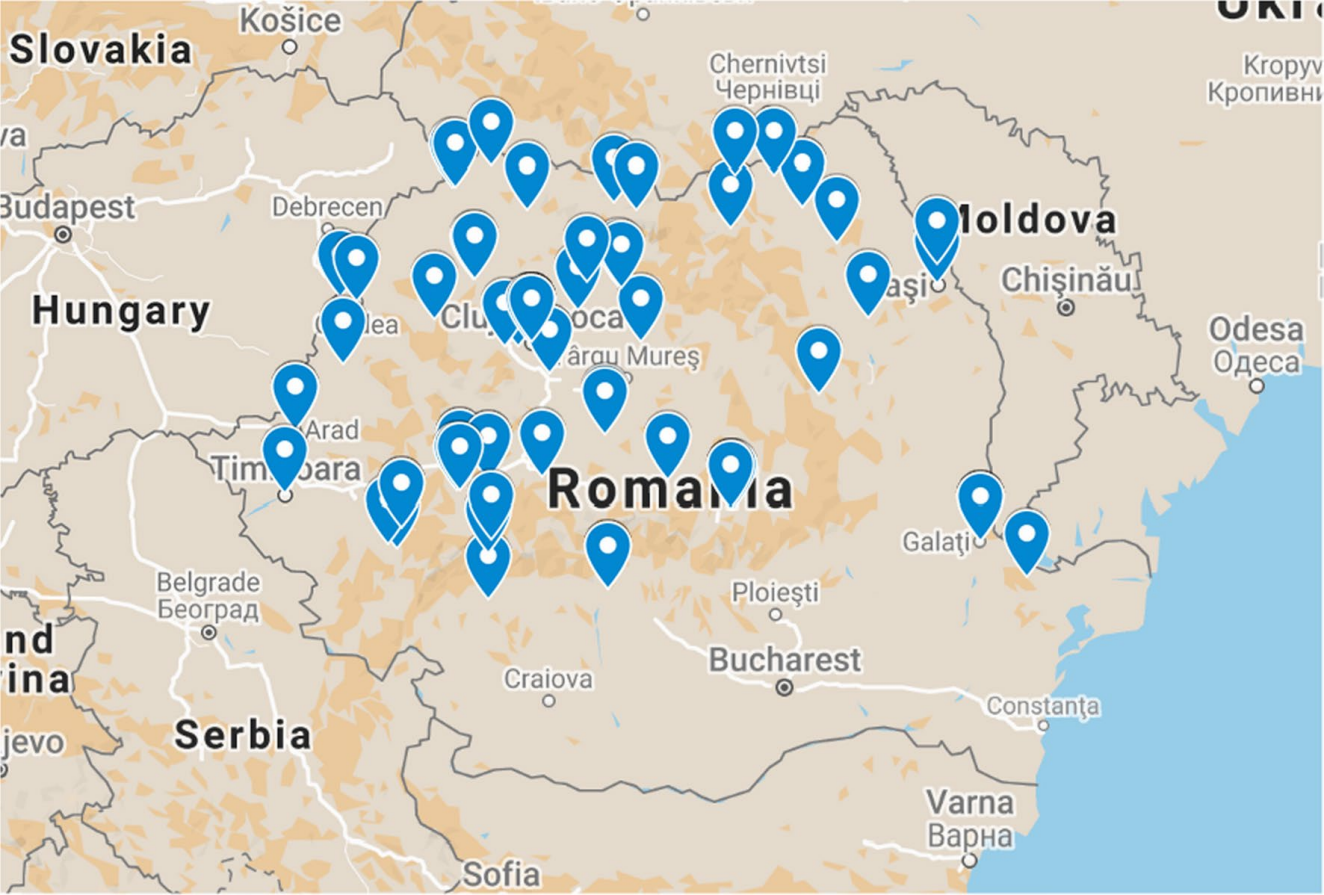
The geographical distribution of patients selected for the study. Map data ©2021 GeoBasis DE/BKG (©2009), Google, Inst. Geogr. Nacional, MapaGIsrael

Patients were selected from various regions of Romania, so that a geographical distribution of the virus in Romania could be studied. The geographical distribution of the patients is shown in Fig. 1.

### PCR results

**Fig. 2:**
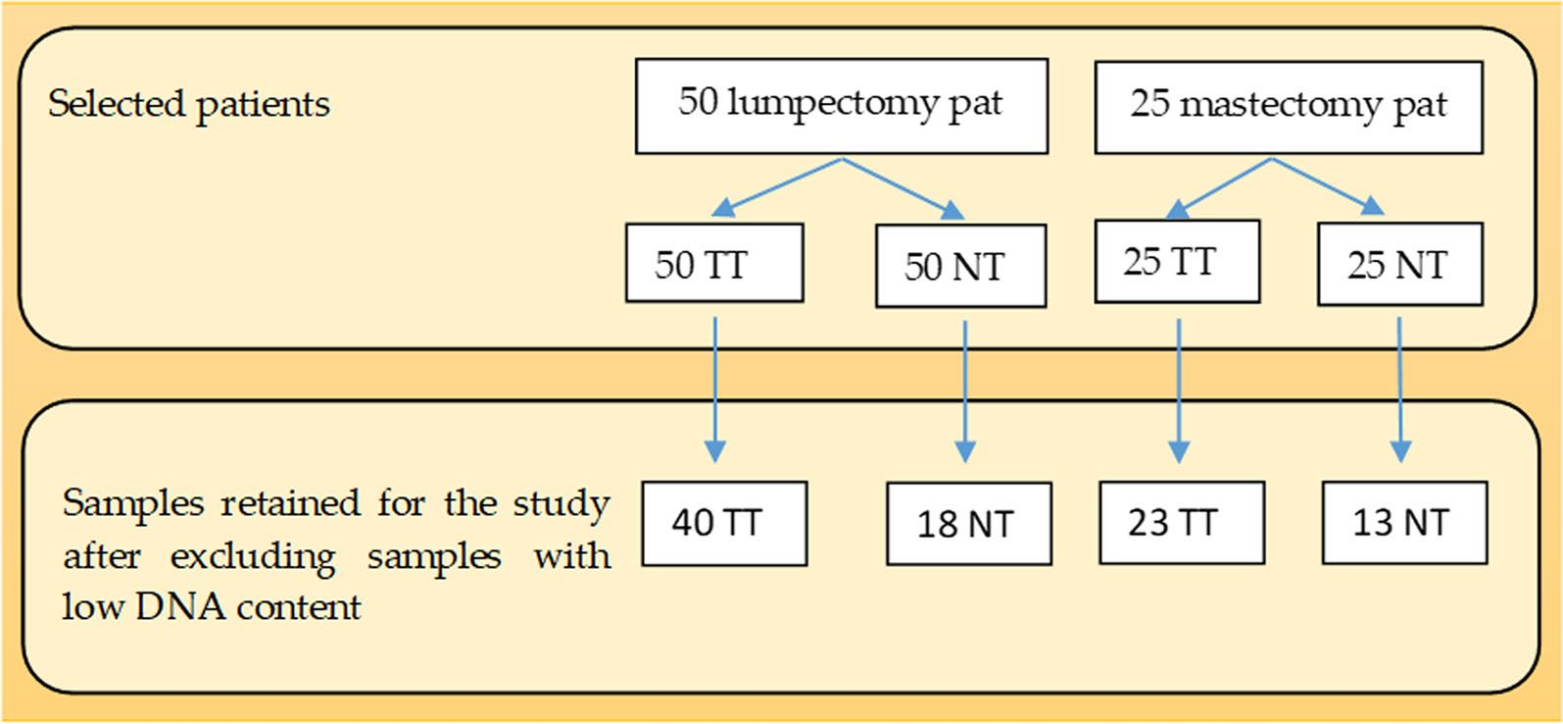
Samples retained for PCR after excluding samples with low tissue and DNA content. *TT* tumour tissue, *NT* normal tissue, *pat* patients

The samples with an insufficient quantity of tissue or a very low concentration of DNA were excluded from the study, as shown in Additional file 1: Tables S1 and S2. More precisely, for some cases, when realizing 10 µm sections for obtaining either tumor or normal tissue we could not obtain at minimum 5 sections for each tube, so these samples had to be excluded. From the lumpectomy group, out of 50 tumor tissue samples, 48 remained for further analysis and out of these 48 samples 40 had sufficient quantity of DNA. Out of 50 normal tissue samples, in 32 cases there was sufficient tissue, but only 18 were kept in the study as samples with proper DNA content. From the mastectomy group, out of 25 tumor tissue samples, 23 had an optimal concentration of DNA, and out of 20 normal tissue samples with sufficient material only 13 were kept in the study for further investigations, for the same reasons. (Fig. 2) Therefore, after DNA extraction, in the lumpectomy group, there were 40 tumor samples and 18 normal tissue samples, while in the mastectomy group there were 23 tumor samples and 13 normal tissue samples.

**None** of the examined samples was positive for MMTV-like target sequences on PCR.

## Discussion

Breast cancer is both an ancient disease, first described by the Egyptians [19], and a disease of civilization, since its incidence has increased in the last century.

In mice, MMTV is possible transmitted in a three-way fashion, having some proof of all three ways of transmissions, as shown below.

1. It might be transmitted **horizontally** as an infectious particle containing viral RNA between related and unrelated individuals. The horizontal transmission of MMTV to unrelated individuals is the least studied way of spread of the virus. Blair et al. [20] showed that spleen reactivity can be induced in isolated female mice by caging them with a mouse neonatally infected with MMTV. Velin et al. [21] successfully infected adult mice with mouse milk containing the viral particles through injection into the nose. MMTV infects the nasal-associated lymphoid tissue (NALT) similar to the infection of Peyer’s plaques.
2. MMTV might be transmitted **vertically in the antenatal period** in the murine germline as an endogenous proviral DNA genome in a Mendelian fashion. MMTV does not contain an oncogene, but it has been shown that proviral-DNA can integrate close to proto-oncogenes and enhance their activity [22].
3. More likely or more frequently MMTV is transmitted **vertically in the post-natal period**. Some authors classify this way of transmission as **horizontal** [23]. MMTV it replicates in the mothers' mammary glands where the infectious B-type particles accumulate in milk. When the nurslings ingest the virus, it invades the gut-associated lymphoid tissues and infects B-lymphocytes. To aid viral replication, MMTV uses a superantigen, encoded by the *orf* gene in the viral long terminal repeat (LTR) region, to activate specific T-lymphocyte subsets. This serves to stimulate viral replication as well as clonal amplification of T lymphocytes, which provides a vehicle for viral passage from the gut-associated lymphatic tissue to the breast. Although viral replication is maximal in the breast epithelium, hormone stimulation is still required to activate the corticosteroid response elements in the retroviral LTR. During pregnancy, viral replication is increased, which leads to hyperplasic nodule formation that can eventually lead to tumor formation [24].

The transmission of MMTV to humans has been studied in numerous publications. The fact that MMTVs can infect human breast cells in culture was demonstrated by Indik et al. [25]*.* Johal et al. [26] found MMTV like virus sequences by PCR in 5% (4/91) of breast milk samples from healthy lactating women from Australia. Furthermore, MMTV-like sequences were found in the saliva, parotid glands [27] and peripheral blood lymphoid cells [28], suggesting that viral transmission in humans is similar to that seen in mice, occurring mostly via breastfeeding and infection of mucosa-associated lymphocytes before reaching the breast tissue. Humans have well developed lymphatic structures in the mouth and nose (tonsils and adenoids), which are possible entry points for MMTV. Faschinger et al. [29] proved that when MMTVs infect human mammary epithelial cells they randomly integrate their genomic information into the human genome of the infected cell.

MMTV can infect different animal species which could possibly spread the infection to humans. MMTV-like gene sequences were found in 20% of mammary tumours in dogs and 33% of mammary tumours in cats [30]. Women with companion dogs are at twice the expected risk of breast cancer, which suggests MMTV could be transmitted in dog saliva to humans [31].

In several countries it is permissible for 1% in weight of cereals to consist of mouse or rat faecal material. Because MMTV is endemic in many *Mus musculus domesticus* populations, transmission by consumption of undercooked cereals and other foods is a hypothesis which has to be taken into account.

What is MMTV’s role in human breast cancer on the globe? Our results are similar with the results of a series of studies that could not detect the presence of the MMTV in human breast cancer samples. Tabriz et al. [17] evaluated the same MMTV-like target sequence as us by RT-PCR using the same primers, and did not detect any virus particle in breast cancer tissues of 40 **Iranian women**. Perzova et al. [32] analyzed the prevalence of MMTV *env* sequences in 66 samples of FFPE (formalin-fixed paraffin-embedded) or snap-frozen biopsies of breast tissue (**US patients**) by PCR and took supplementary measures to prevent carry-over contamination with murine DNA; they concluded that the MMTV copy numbers detected in the breast cancer specimens were too low to be derived from a monoclonally integrated expanded tumor cell sample. Fukuoka et al. [33] investigated **in Japan** 46 breast cancer patients and 3 patients with benign mammary tumors and used PCR and Southern blot hybridization; their team could not detect the MMTV *env* gene-like sequence in any of the samples tested.

However, there are several reports on the MMTV *env* gene in human breast cancer samples [15, 34–36].

The contrast between the different outcomes of the studies indicates that there is a regional virus epidemiology. A comparative analysis was undertaken by Levine et al. [37] While looking for the same 250-bp sequence using the same amplification techniques, the group showed the highest percentage of MMTV-like sequences, 74% in Tunisia, intermediate incidence (31–42.2%) in the U.S., Western-Europe, Australia and Argentina, and only 0.8% in Vietnam.

Our study is the first one in Romania and even South-Eastern Europe, which analyzes the prevalence of MMTV-like viral sequences in human breast tissue. Based on our sample the results indicate that breast cancer is not likely to be related to the MMTV infection in Romania. This result is most likely explained by the transmission vectors of the virus and the geographic distribution of *Mus musculus sp. domesticus*—the native MMTV reservoir. It has been proposed that a higher prevalence in some areas of *Mus musculus domesticus* in the vicinity of humans, which is the species of mouse that carries the infectious MMTV, is correlated to a higher prevalence of human breast cancer [38]. *M. m. domesticus* seems to be less present in South-Eastern Europe, where *M. m. musculus* is the predominant species [39]. In relationship to MMTV this variability of species needs to be further explored, but we found no reports of naturally occurring MMTV infection in *M. m. musculus*. Callahan et al. [40] studying several feral mouse species stated that *M. m. musculus* specimens from Czechoslovakia did not carry MMTV, although all mice species studied had small fragments of inserted MMTV which is explained by the “accumulation of evolutionarily divergent MMTV-alpha insertions into the mouse germ line”. Ford et al. [35] could not identify MMTV in first-generation Australian-Vietnamese women, in comparison to Caucasian-Australian woman (42.2%). It is known that in South-Eastern Asia the dominant mouse species is not *M. m. domesticus*, but *M. m. castaneus*.

Recently, Wang et al. [41] described a regional distribution of MMTV-positive breast tissue specimens in China: in Southern China the positivity was 5.71%, compared to 22.62% in Northern China (*p* < 0.05). This different prevalence is likely to be linked to the distribution of *M. m. domesticus* and *M. m. castaneus.* Stewart et al. [42] presented evidence that mouse population outbreaks are correlated with spikes in breast cancer incidence in Australia and New Zealand.

Furthermore, even *Mus musculus sp. domesticus*, also known as “western house mouse”, since its spread to Europe from Asia in the postglacial period [38], clustered in somewhat different local populations [39], and local subpopulations may have different rates of infection with MMTV, which might explain the variable positivity of MMTV in Western countries, ranging 20–40%. Another reason for the variability of the rate of association of MMTV to human breast cancer is the presence of a possible intermediary host, the dog. Laumbacher et al. [31] studied dog–human contact epidemiologically in Bavaria, Germany and found that more than twice the number of breast cancer patients kept dogs permanently in the last 10 years leading up to the diagnosis compared to control individuals (37.7% vs. 14.8%, *p* = 0.0000003, relative risk 3.5).

Because breast cancer is more prevalent among women of higher socio-economic status, it has been hypothesized that this may be due to a late exposure to MMTV infection from mice/dogs, as compared to infection during early life among girls of low socio-economic status and hence early immunity [43].

As human breast cancer progresses, the MMTV viral load increases but it falls in late-stage invasive breast cancer [16]. The lack of identification of MMTV in advanced breast cancer may be due to the breakdown of cell physiology, but our patient sample consisted of early stage, non-metastatic breast cancer patients.

Another explanation for the divergent results regarding the MMTV infection in humans might be that some positive results were due to carry-over contamination of human samples with previously amplified murine MMTV DNA. Rodent DNA might be present in the building’s walls and ventilation systems, most likely as small particulate matter [32]. We consider this scenario very unlikely in most of the MMTV-positive studies, although might have had happened in some, taking into account the extreme measures taken to avoid contamination by most of the laboratories.

While it is clear that the association between MMTV infection and human breast cancer is still a debatable topic, most results indicate that a correlation exists. Proving that MMTV is an etiologic agent for human breast cancer will probably require non-PCR based traditional retrovirology techniques such as virus isolation and determination of monoclonal integration in tumor cells. This would certainly be a breakthrough in oncology and would open the gates to the prevention of breast cancer through (1) hygiene measures, (2) counseling of those less than 5% of MMTV-infected women to find alternatives to breastfeeding and (3) the development of a vaccine. If in Western countries and Australia the involvement of MMTV/MMTV-like viruses in breast cancer probably occurs in 20–40% of patients, such measures would lead to a proportional reduction of newly diagnosed breast cancer cases in about one or two generations.

Other virus infections have been proposed as well as causative agents of breast cancer; the most studied is bovine leukemia virus (BLV) [44].

Breast cancer remains one of the most mysterious human cancers, since most cases cannot be linked to specific risk factors.

We could not conclude our discussion without adding a cautionary note that our results should be confirmed by other alternative virology assays and other laboratories and international collaboration is needed to further clarify the involvement of MMTV or MMTV-like viruses in breast cancer.

## Conclusions

We have observed that MMTV-like sequences are not present in our region in breast tissue samples. The same result was noted by other geographically related research groups. Only *Mus musculus domesticus* mouse species was proven to carry infectious MMTV, thus our results might be related to the variable local distribution of different mice species (*M. m. domesticus*, *musculus* and *castaneus*) and the historical/current infection pattern with the virus.

## Supplementary Information

**Additional file 1**. Sample selection

## Data Availability

All data supporting the findings of this study are available with the corresponding authors. The data is not publicly available, since it could compromise the privacy of the research participants.

## Abbreviations

MMTV: Mouse mammary tumor virus
PCR: Polymerase chain reaction
FFPE: Formalin-fixed paraffin-embedded
NT: Normal tissue
TT: Tumor tissue
