# Supplementary material for: Prevalence of MMTV-like sequences in breast cancer samples in Romanian patients-there is a geographic difference compared to the Western world
Source: Infect Agent Cancer. 2023 Jun 20;18:39. doi: 10.1186/s13027-023-00486-y (PMC10283304; doi:10.1186/s13027-023-00486-y)
Supplement: Supplementary file 1 — Additional file 1. Sample selection [file 13027_2023_486_MOESM1_ESM.docx]

Supplementary Materials:

**Table S1**: Mastectomy samples, DNA extraction results.

Abbreviations: TT=tumoural tissue, TN=normal tissue.

| **#** | **Sample**  **ID** | **DNA Conc.** |  | **Unit** | **260/280** | **260/230** | **DNA (200ng)** | **H2O** |
| --- | --- | --- | --- | --- | --- | --- | --- | --- |
| 1 | 1M TN | 2 |  | ng/µl | 1.36 | 0.87 | 100.00 | -76.00 |
| 2 | 2M TN | 5.8 |  | ng/µl | 1.55 | 1.38 | 34.48 | -10.48 |
| 3 | 3M TN | 39 |  | ng/µl | 1.68 | 2.26 | 5.13 | 18.87 |
| 4 | 4M TN | 0.2 |  | ng/µl | -2.53 | -0.05 | 1000.00 | -976.00 |
| 5 | 6M TN | 9.7 |  | ng/µl | 1.53 | 1.19 | 20.62 | 3.38 |
| 6 | 7M TN | -1.5 |  | ng/µl | 3.79 | 0.18 | -133.33 | 157.33 |
| 7 | 8M TN | 6.4 |  | ng/µl | 1.69 | 1.66 | 31.25 | -7.25 |
| 8 | 9M TN | 44.9 |  | ng/µl | 1.71 | 2.57 | 4.45 | 19.55 |
| 9 | 10M TN | 15.9 |  | ng/µl | 1.72 | 2.59 | 12.58 | 11.42 |
| 10 | 11M TN | 1.1 |  | ng/µl | 1.81 | -0.39 | 181.82 | -157.82 |
| 11 | 12M TN | 17.9 |  | ng/µl | 1.8 | 3.4 | 11.17 | 12.83 |
| 12 | 15M TN | 5.9 |  | ng/µl | 1.91 | -16.54 | 33.90 | -9.90 |
| 13 | 16M TN | 17 |  | ng/µl | 1.75 | 1.05 | 11.76 | 12.24 |
| 14 | 17M TN | 33.1 |  | ng/µl | 1.81 | 2.68 | 6.04 | 17.96 |
| 15 | 18M TN | 54.5 |  | ng/µl | 1.75 | 2.28 | 3.67 | 20.33 |
| 16 | 19M TN | 11.6 |  | ng/µl | 1.72 | 2.34 | 17.24 | 6.76 |
| 17 | 21M TN | 46.7 |  | ng/µl | 1.76 | 2.5 | 4.28 | 19.72 |
| 18 | 23M TN | 9.2 |  | ng/µl | 1.56 | 0.75 | 21.74 | 2.26 |
| 19 | 24M TN | 8.7 |  | ng/µl | 1.55 | 1.76 | 22.99 | 1.01 |
| 20 | 25M TN | 20.6 |  | ng/µl | 1.71 | 2.73 | 9.71 | 14.29 |
|  |  |  |  |  |  |  |  |  |
| 1 | 1M TT | 25.2 |  | ng/µl | 1.48 | 0.59 | 7.94 | 16.06 |
| 2 | 2M TT | 3.9 |  | ng/µl | 1.63 | 22.87 | 51.28 | -27.28 |
| 3 | 3M TT | 390.5 |  | ng/µl | 1.81 | 2.14 | 0.51 | 23.49 |
| 4 | 4M TT | 2.9 |  | ng/µl | 1.23 | -1.35 | 68.97 | -44.97 |
| 5 | 5M TT | 11.1 |  | ng/µl | 1.79 | 2.78 | 18.02 | 5.98 |
| 6 | 6M TT | 6.9 |  | ng/µl | 1.48 | 0.81 | 28.99 | -4.99 |
| 7 | 7M TT | 94.1 |  | ng/µl | 1.76 | 1.62 | 2.13 | 21.87 |
| 8 | 8M TT | 25.9 |  | ng/µl | 1.73 | 3 | 7.72 | 16.28 |
| 9 | 9M TT | 90.2 |  | ng/µl | 1.72 | 2 | 2.22 | 21.78 |
| 10 | 10M TT | 39.6 |  | ng/µl | 1.63 | 1.64 | 5.05 | 18.95 |
| 11 | 11M TT | 234.1 |  | ng/µl | 1.67 | 2.15 | 0.85 | 23.15 |
| 12 | 12M TT | 566.6 |  | ng/µl | 1.81 | 2.15 | 0.35 | 23.65 |
| 13 | 13M TT | 158.4 |  | ng/µl | 1.77 | 1.66 | 1.26 | 22.74 |
| 14 | 14M TT | 698 |  | ng/µl | 1.79 | 1.92 | 0.29 | 23.71 |
| 15 | 15M TT | 79 |  | ng/µl | 1.77 | 2.13 | 2.53 | 21.47 |
| 16 | 16M TT | 32.5 |  | ng/µl | 1.7 | 1.84 | 6.15 | 17.85 |
| 17 | 17M TT | 10.1 |  | ng/µl | 1.81 | 6.42 | 19.80 | 4.20 |
| 18 | 18M TT | 190.7 |  | ng/µl | 1.76 | 1.98 | 1.05 | 22.95 |
| 19 | 19M TT | 102.6 |  | ng/µl | 1.72 | 2.03 | 1.95 | 22.05 |
| 20 | 20M TT | 44.7 |  | ng/µl | 1.75 | 2.54 | 4.47 | 19.53 |
| 21 | 21M TT | 46 |  | ng/µl | 1.73 | 2.21 | 4.35 | 19.65 |
| 22 | 22M TT | 399.6 |  | ng/µl | 1.77 | 2.05 | 0.50 | 23.50 |
| 23 | 23M TT | 11.7 |  | ng/µl | 1.64 | 1.2 | 17.09 | 6.91 |
| 24 | 24M TT | 24.1 |  | ng/µl | 1.76 | 2.09 | 8.30 | 15.70 |
| 25 | 25M TT | 70.8 |  | ng/µl | 1.69 | 1.74 | 2.82 | 21.18 |

**Table S2:** Lumpectomy samples, DNA extraction results.

Abbreviations: TT=tumoural tissue, TN=normal tissue.

| # | **Sample**  **ID** | **ADN Conc.** | **Unit** | **260/280** | **260/230** | **DNA**  **(200 ng)** | **H2O** |
| --- | --- | --- | --- | --- | --- | --- | --- |
| 1 | 1S TN | 49.4 | ng/µl | 1.71 | 2.07 | 4.05 | 19.95 |
| 2 | 2S TN | 100.2 | ng/µl | 1.76 | 2.42 | 2.00 | 22.00 |
| 3 | 3S TN | -0.9 | ng/µl | -0.63 | 0.3 | -222.22 | 246.22 |
| 4 | 4S TN | 16 | ng/µl | 1.53 | 3.88 | 12.50 | 11.50 |
| 5 | 6S TN | 3.2 | ng/µl | 0.89 | 19.84 | 62.50 | -38.50 |
| 6 | 7S TN | 3.5 | ng/µl | 0.84 | -2.58 | 57.14 | -33.14 |
| 7 | 8S TN | 7 | ng/µl | 1.16 | -1804.79 | 28.57 | -4.57 |
| 8 | 12S TN | 1.2 | ng/µl | 0.47 | -0.61 | 166.67 | -142.67 |
| 9 | 13S TN | 12.6 | ng/µl | 1.44 | 3.05 | 15.87 | 8.13 |
| 10 | 14S TN | 0.1 | ng/µl | 0.06 | -0.05 | 2000.00 | -1976.00 |
| 11 | 16S TN | 28.2 | ng/µl | 1.69 | 2.96 | 7.09 | 16.91 |
| 12 | 19S TN | 152 | ng/µl | 1.78 | 2.24 | 1.32 | 22.68 |
| 13 | 20S TN | 22.4 | ng/µl | 1.55 | 2.02 | 8.93 | 15.07 |
| 14 | 22S TN | -0.6 | ng/µl | -0.31 | -0.07 | -333.33 | 357.33 |
| 15 | 25S TN | -2.2 | ng/µl | -4.05 | 0.78 | -90.91 | 114.91 |
| 16 | 26S TN | 2.7 | ng/µl | 0.77 | -1.51 | 74.07 | -50.07 |
| 17 | 29S TN | 128.2 | ng/µl | 1.77 | 2.64 | 1.56 | 22.44 |
| 18 | 30S TN | 2.8 | ng/µl | 1.49 | -0.64 | 71.43 | -47.43 |
| 19 | 31S TN | 1.4 | ng/µl | 2.05 | -0.2 | 142.86 | -118.86 |
| 20 | 32S TN | 5.2 | ng/µl | 1.82 | -1.07 | 38.46 | -14.46 |
| 21 | 34S TN | 6.9 | ng/µl | 1.63 | 19.18 | 28.99 | -4.99 |
| 22 | 35S TN | 34.1 | ng/µl | 1.68 | 3.35 | 5.87 | 18.13 |
| 23 | 38S TN | 4.7 | ng/µl | 1.83 | -0.81 | 42.55 | -18.55 |
| 24 | 39S TN | 6.8 | ng/µl | 1.74 | -2.63 | 29.41 | -5.41 |
| 25 | 40S TN | 15.6 | ng/µl | 1.71 | -17.58 | 12.82 | 11.18 |
| 26 | 41S TN | 1.5 | ng/µl | 1.74 | -0.21 | 133.33 | -109.33 |
| 27 | 42S TN | 136.2 | ng/µl | 1.56 | 2.33 | 1.47 | 22.53 |
| 28 | 43S TN | 15.1 | ng/µl | 1.56 | 11.59 | 13.25 | 10.75 |
| 29 | 46S TN | 5.7 | ng/µl | 1.87 | -1.22 | 35.09 | -11.09 |
| 30 | 47S TN | 8.6 | ng/µl | 1.69 | -2.2 | 23.26 | 0.74 |
| 31 | 48S TN | 169.5 | ng/µl | 1.65 | 2.31 | 1.18 | 22.82 |
| 32 | 50S TN | 12.6 | ng/µl | 1.82 | -7.08 | 15.87 | 8.13 |
|  |  |  |  |  |  |  |  |
| 1 | 1S TT | 120.7 | ng/µl | 1.69 | 2.28 | 1.66 | 22.34 |
| 2 | 2S TT | 107.2 | ng/µl | 1.72 | 2.21 | 1.87 | 22.13 |
| 3 | 3S TT | 5.9 | ng/µl | 1.75 | -19.58 | 33.90 | -9.90 |
| 4 | 4S TT | 91.9 | ng/µl | 1.69 | 2.4 | 2.18 | 21.82 |
| 5 | 5S TT | 23.2 | ng/µl | 1.89 | 2.78 | 8.62 | 15.38 |
| 6 | 6S TT | 43.7 | ng/µl | 1.76 | 1.87 | 4.58 | 19.42 |
| 7 | 7S TT | 7.3 | ng/µl | 1.81 | 2.89 | 27.40 | -3.40 |
| 8 | 8S TT | 24.8 | ng/µl | 1.57 | 1.33 | 8.06 | 15.94 |
| 9 | 9S TT | 9.5 | ng/µl | 1.61 | 1.41 | 21.05 | 2.95 |
| 10 | 10S TT | 6.1 | ng/µl | 1.74 | 0.87 | 32.79 | -8.79 |
| 11 | 11S TT | 131.3 | ng/µl | 1.76 | 2.03 | 1.52 | 22.48 |
| 12 | 13S TT | 25.7 | ng/µl | 1.8 | 2.38 | 7.78 | 16.22 |
| 13 | 14S TT | 10.3 | ng/µl | 1.81 | 1.33 | 19.42 | 4.58 |
| 14 | 15S TT | 13 | ng/µl | 1.82 | 2.66 | 15.38 | 8.62 |
| 15 | 16S TT | 71.5 | ng/µl | 1.8 | 2.41 | 2.80 | 21.20 |
| 16 | 17S TT | 38.6 | ng/µl | 1.77 | 2.62 | 5.18 | 18.82 |
| 17 | 18S TT | 12.4 | ng/µl | 1.84 | 2.42 | 16.13 | 7.87 |
| 18 | 19S TT | 10.7 | ng/µl | 1.89 | 2.22 | 18.69 | 5.31 |
| 19 | 20S TT | 24.6 | ng/µl | 1.84 | 2.79 | 8.13 | 15.87 |
| 20 | 21S TT | 6.1 | ng/µl | 1.68 | 1.15 | 32.79 | -8.79 |
| 21 | 22S TT | 25.9 | ng/µl | 1.73 | 2.02 | 7.72 | 16.28 |
| 22 | 23S TT | 17.5 | ng/µl | 1.96 | 2.9 | 11.43 | 12.57 |
| 23 | 24S TT | 31.3 | ng/µl | 1.76 | 2.41 | 6.39 | 17.61 |
| 24 | 25S TT | 28.1 | ng/µl | 1.63 | 1.04 | 7.12 | 16.88 |
| 25 | 26S TT | 23.4 | ng/µl | 1.7 | 2.52 | 8.55 | 15.45 |
| 26 | 27S TT | 12.3 | ng/µl | 1.78 | 2.16 | 16.26 | 7.74 |
| 27 | 28S TT | 5.1 | ng/µl | 1.6 | 2.6 | 39.22 | -15.22 |
| 28 | 29S TT | 11.1 | ng/µl | 1.91 | 3.5 | 18.02 | 5.98 |
| 29 | 30S TT | 4.2 | ng/µl | 1.94 | 3.28 | 47.62 | -23.62 |
| 30 | 31S TT | 13.1 | ng/µl | 1.71 | 2.1 | 15.27 | 8.73 |
| 31 | 32S TT | 15.2 | ng/µl | 1.79 | 2.25 | 13.16 | 10.84 |
| 32 | 33S TT | 68.4 | ng/µl | 1.65 | 0.9 | 2.92 | 21.08 |
| 33 | 34S TT | 5 | ng/µl | 1.49 | 1.75 | 40.00 | -16.00 |
| 34 | 35S TT | 7.3 | ng/µl | 1.45 | 0.67 | 27.40 | -3.40 |
| 35 | 36S TT | 10.6 | ng/µl | 1.79 | 3.5 | 18.87 | 5.13 |
| 36 | 37S TT | 4.1 | ng/µl | 1.69 | 84.43 | 48.78 | -24.78 |
| 37 | 38S TT | 27.4 | ng/µl | 1.74 | 2.73 | 7.30 | 16.70 |
| 38 | 40S TT | 13.6 | ng/µl | 1.64 | 2.33 | 14.71 | 9.29 |
| 39 | 41S TT | 92.4 | ng/µl | 1.63 | 2.36 | 2.16 | 21.84 |
| 40 | 42S TT | 60.2 | ng/µl | 1.55 | 2.3 | 3.32 | 20.68 |
| 41 | 43S TT | 45.6 | ng/µl | 1.57 | 2.32 | 4.39 | 19.61 |
| 42 | 44S TT | 0.6 | ng/µl | 0.94 | -0.2 | 333.33 | -309.33 |
| 43 | 45S TT | 9.7 | ng/µl | 1.62 | 6.66 | 20.62 | 3.38 |
| 44 | 46S TT | 18 | ng/µl | 1.65 | 3.66 | 11.11 | 12.89 |
| 45 | 47S TT | 223.4 | ng/µl | 1.58 | 1.64 | 0.90 | 23.10 |
| 46 | 48S TT | 58.8 | ng/µl | 1.58 | 2.11 | 3.40 | 20.60 |
| 47 | 49S TT | 29.4 | ng/µl | 1.64 | 1.89 | 6.80 | 17.20 |
| 48 | 50S TT | 31.4 | ng/µl | 1.67 | 2.33 | 6.37 | 17.63 |
